# Crystal structure of (2*E*)-2-meth­oxy­imino-2-{2-[(2-methyl­phen­oxy)meth­yl]phen­yl}-*N*′-(4-nitro­benzyl­idene)ethano­hydrazide

**DOI:** 10.1107/S2056989015004569

**Published:** 2015-03-18

**Authors:** Chetan Shrimandhar Shripanavar, Ray J. Butcher

**Affiliations:** a P.G. Department of Agrochemicals and Pest Management, Devchand College, Arjunnagar 591 237, M.S., India; b Department of Chemistry, Howard University, 525 College Street NW, Washington, DC 20059, USA

**Keywords:** crystal structure, kresoxim-methyl derivatives, broad spectrum fungicides, hydrogen bonding

## Abstract

The title compound, C_24_H_22_N_4_O_5_, crystallizes with two mol­ecules in the asymmetric unit (*Z*′ = 2) oriented almost perpendicular to each other [dihedral angle between the central core of each mol­ecule = 77.95 (3)°]. The two mol­ecules exhibit similar conformations with an extended structure. An intra­molecular C—H⋯N hydrogen bond occurs in each mol­ecule. The two mol­ecules are linked by a bifurcated N—H⋯(O,N) hydrogen bond involving the NH group in mol­ecule *A* as donor. They are further linked into a ribbon along the *a*-axis direction by further bifurcated N—H⋯(O,N) hydrogen bonds involving the NH group in mol­ecule *B* as donor. C—H⋯O inter­actions are also observed.

## Abstract 2

The title compound crystallizes with the two mol­ecules in the asymmetric unit (*Z*′ = 2) oriented almost perpendicular to each other [dihedral angle between the central core of each mol­ecule = 77.95 (3)°]. The two mol­ecules exhibit similar conformations with an extended structure and are linked by bifurcated hydrogen bonds into a ribbon along the *a*-axis direction.

## Chemical context   

Kresoxim-methyl [systematic name: methyl (2*E*)-(meth­oxy­imino){2-[(2-methyl­phen­oxy)meth­yl]phen­yl}acetate] derivatives are broad spectrum fungicides (Anke *et al.*, 1977 ▸), have a site-specific action (Olaya *et al.*, 1998 ▸) and exhibit high efficiency (Patel *et al.*, 2012 ▸; Esteve-Turrillas *et al.*, 2011 ▸; Mercader *et al.*, 2008 ▸) against various diseases of agricultural crops (Balba, 2007 ▸; Cash & Cronan, 2001 ▸; Ammermann *et al.*, 2000 ▸). As these types of compounds are easily metabolized in nature as well as in living systems, their modifications are of immense importance (Balba, 2007 ▸). In order to increases the activity of starting compounds (Kant *et al.*, 2012 ▸), it is necessary to modify their structures and to undertake a structural investigation of different kresoxim-methyl derivatives.
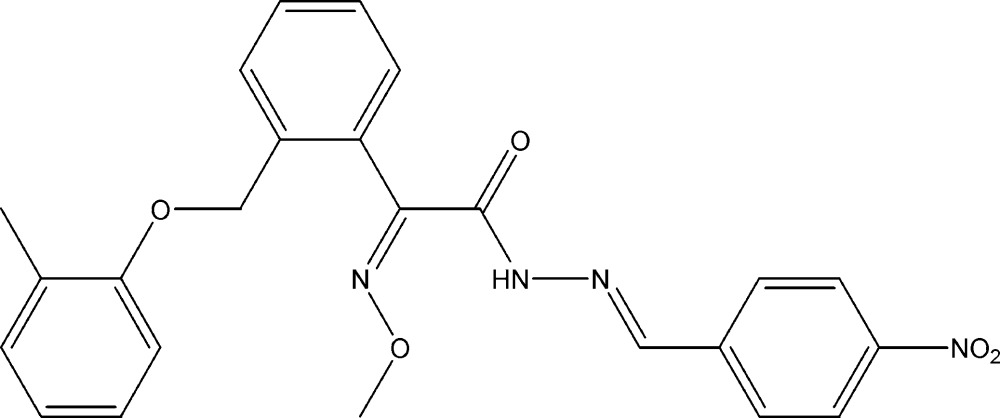


## Structural commentary   

**Figure 1:**
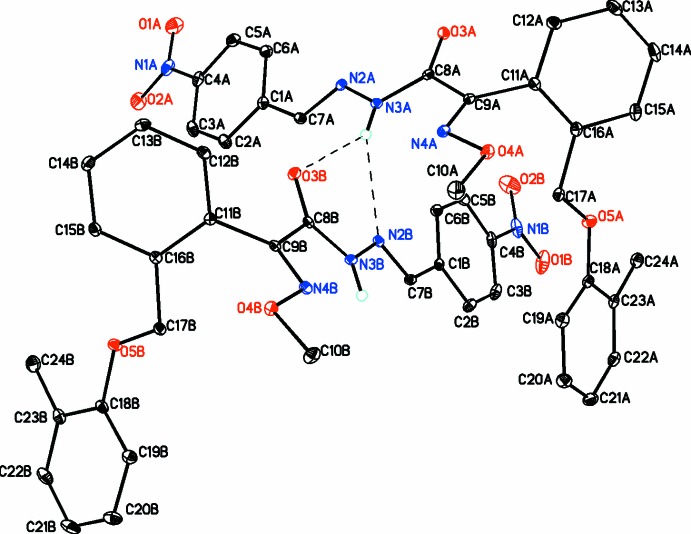
The mol­ecular structure of mol­ecules *A* and *B* of the title compound, showing the atom labeling and displacement ellipsoids at the 30% probability level. Hydrogen bonds are shown as dashed lines. All H atoms, except those involved in hydrogen bonding, have been omitted for clarity.

The title compound crystallizes with two mol­ecules in the asymmetric unit (*Z*′ = 2) labeled *A* and *B* and shown in Fig. 1 ▸. The two mol­ecules exhibit similar conformations having an extended structure. In mol­ecule *A*, the nitro group is coplanar with the *p*-nitro­phenyl ring [deviations for N1*A*, O1*A* and O2*A* of 0.067 (2), 0.119 (2) and 0.089 (2) Å, respectively]. The central ethane hydrazide moiety (N2*A*/N3*A*/C8*A*/O3*A*) is strictly planar with an r.m.s. deviation of 0.000 Å for the fitted atoms. The dihedral angles between this moiety and the adjacent aromatic are 18.99 (4)° for the nitro­benzylidene ring (C1*A*–C6*A*) and 62.20 (4)° for the benzene ring (C11*A*–C16*A*).

In mol­ecule *B*, the nitro group is coplanar with the *p*-nitro­phenyl ring [deviations for N1*B*, O1*B* and O2*B* of 0.026 (2), 0.043 (2) and 0.127 (2) Å, respectively]. The central ethane hydrazide moiety (N2*B*/N3*B*/C8*B*/O3*B*) is planar (r.m.s. deviation = 0.002 Å). The dihedral angles between this moiety and the adjacent aromatic rings are 12.43 (4)° for the nitrobenzylidene ring (C1*B*–C6*B*) and 57.99 (4)° for the benzene ring (C11*B*–C16*B*).

Mol­ecules *A* and *B* are oriented almost perpendicular to each other, the dihedral angle between their central cores (atoms C7 N2 N3 and C8) being 77.95 (3)°.

For both mol­ecules, bond lengths and angles are all within the normal ranges; however, comparisons with similar mol­ecules cannot be made as there are no similar overall structures although, of course, their fragments exist.

An intra­molecular hydrogen bond (C17*A*—H17*B*⋯N4*A* and C17*B*—H17*C*⋯N4*B*; Table 1 ▸) occurs in each independent mol­ecule.

## Supra­molecular features   

**Figure 2:**
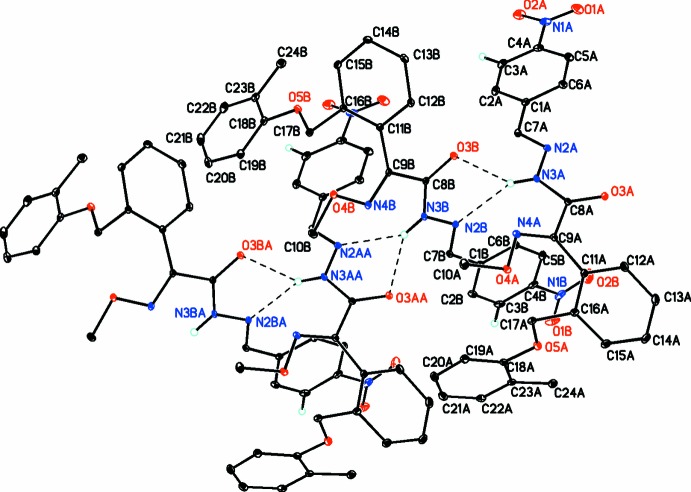
Diagram showing the two mol­ecules (*A* and *B*) linked by a bifurcated hydrogen bond between the N3*A*—H3*A* group and atoms O3*B* and N2*B*, and further linked into a ribbon along the *a*-axis direction by a bifurcated hydrogen bond between the N3*B*—H3*B* group and atoms O3*A* and N2*A* (generated by the symmetry operation *x* + 1, *y*, *z*).

The two independent mol­ecules are linked by a bifurcated hydrogen bond (Table 1 ▸) between N3*A*—H3*A*⋯(O3*B*,N2*B*). The mol­ecules are further linked into a ribbon along the *a*-axis direction a bifurcated N3*B*—H3*B*⋯(O3*A*,N2*A*)(*x* + 1, *y*, *z*) hydrogen bond involving the corresponding NH group in the other independent mol­ecule, as shown in Fig. 2 ▸. C—H⋯O inter­actions link the ribbons into a three-dimensional array.

## Database survey   

A search of the Cambridge Structural Database (CSD, Version 5.35, last update November 2014; Groom & Allen, 2014 ▸) for the basic skeleton of this compound gave no hits.

## Synthesis and crystallization   

(2*E*)-2-Meth­oxy­imino-2-{2-[(2-methyl­phen­oxy)meth­yl]phenyl}ethane­hydrazide (3.13 g, 0.01 mol) was refluxed with *p*-nitro­benzaldehyde (1.51 g, 0.01 mol) in the presence of 5 drops of glacial acetic acid in 20 ml absolute ethanol for about 10 h to get a white-colored product. This was dissolved in methanol and white crystals were obtained by slow evaporation.

## Refinement   

Crystal data, data collection and structure refinement details are summarized in Table 2 ▸. Amine H atoms were refined isotropically. All other H atoms were positioned geometric­ally and refined as riding: C—H = 0.93–0.99 Å with *U_iso_*(H) = 1.5*U*_eq_(C) for methyl H atoms and = 1.2*U_eq_*(C) for other H atoms.

## Supplementary Material

Crystal structure: contains datablock(s) I. DOI: 10.1107/S2056989015004569/hg5434sup1.cif

Structure factors: contains datablock(s) I. DOI: 10.1107/S2056989015004569/hg5434Isup2.hkl

Supporting information file. DOI: 10.1107/S2056989015004569/hg5434Isup3.cml

CCDC reference: 1052231

Additional supporting information:  crystallographic information; 3D view; checkCIF report

## Figures and Tables

**Table 1:** Hydrogen-bond geometry (, )

*D*H*A*	*D*H	H*A*	*D* *A*	*D*H*A*
N3*A*H3*A*O3*B*	0.871(17)	2.117(17)	2.8679(13)	144.0(14)
N3*A*H3*A*N2*B*	0.871(17)	2.432(16)	3.1530(14)	140.5(13)
C7*A*H7*AA*O3*B*	0.95	2.47	3.1523(14)	129
C10*A*H10*C*O1*B* ^i^	0.98	2.46	3.3159(18)	145
N3*B*H3*B*O3*A* ^ii^	0.901(16)	2.068(16)	2.8605(13)	146.1(13)
N3*B*H3*B*N2*A* ^ii^	0.901(16)	2.444(15)	3.1598(14)	136.6(12)
C5*B*H5*BA*O2*A* ^iii^	0.95	2.63	3.3484(18)	133
C7*B*H7*BA*O3*A* ^ii^	0.95	2.51	3.1864(14)	128
C17*B*H17*C*N4*B*	0.99	2.63	3.2436(15)	120

Symmetry codes: (i) 
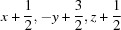
; (ii) 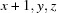; (iii) 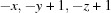.

**Table 2:** Experimental details

Crystal data	
Chemical formula	C_24_H_22_N_4_O_5_
*M*_r_	446.45
Crystal system, space group	Monoclinic, *P*2_1_/*n*
Temperature (K)	120
*a*, *b*, *c* ()	7.6821(4), 23.2151(12), 25.1943(15)
()	95.803(2)
*V* (^3^)	4470.1(4)
*Z*	8
Radiation type	Mo *K*
(mm^1^)	0.10
Crystal size (mm)	0.45 0.21 0.14

Data collection	
Diffractometer	Bruker APEXII
Absorption correction	Multi-scan (*SADABS*; Sheldrick, 1996 ▸)
*T*_min_, *T*_max_	0.692, 0.746
No. of measured, independent and observed [*I* > 2(*I*)] reflections	34631, 11046, 8755
*R*_int_	0.031
(sin /)_max_ (^1^)	0.668

Refinement	
*R*[*F* ^2^ > 2(*F* ^2^)], *wR*(*F* ^2^), *S*	0.040, 0.094, 1.02
No. of reflections	11046
No. of parameters	607
H-atom treatment	H atoms treated by a mixture of independent and constrained refinement
_max_, _min_ (e ^3^)	0.35, 0.22

Computer programs: *APEX2* and *SAINT* (Bruker, 2005 ▸), *SUPERFLIP* (Palatinus Chapuis 2007 ▸), *SHELXL2014* (Sheldrick, 2015 ▸) and *SHELXTL* (Sheldrick, 2008 ▸).

## supplementary crystallographic information

### Crystal data

**Table:**

C_24_H_22_N_4_O_5_	*F*(000) = 1872
*M**_r_* = 446.45	*D*_x_ = 1.327 Mg m^−^^3^
Monoclinic, *P*2_1_/*n*	Mo *K*α radiation, λ = 0.71073 Å
*a* = 7.6821 (4) Å	Cell parameters from 9882 reflections
*b* = 23.2151 (12) Å	θ = 2.4–28.2°
*c* = 25.1943 (15) Å	µ = 0.10 mm^−^^1^
β = 95.803 (2)°	*T* = 120 K
*V* = 4470.1 (4) Å^3^	Needle, colourless
*Z* = 8	0.45 × 0.21 × 0.14 mm

### Data collection

**Table:**

Bruker APEXII diffractometer	8755 reflections with *I* > 2σ(*I*)
ω scans	*R*_int_ = 0.031
Absorption correction: multi-scan (*SADABS*; Sheldrick, 1996)	θ_max_ = 28.3°, θ_min_ = 2.4°
*T*_min_ = 0.692, *T*_max_ = 0.746	*h* = −10→10
34631 measured reflections	*k* = −30→30
11046 independent reflections	*l* = −33→33

### Refinement

**Table:**

Refinement on *F*^2^	0 restraints
Least-squares matrix: full	Hydrogen site location: mixed
*R*[*F*^2^ > 2σ(*F*^2^)] = 0.040	H atoms treated by a mixture of independent and constrained refinement
*wR*(*F*^2^) = 0.094	*w* = 1/[σ^2^(*F*_o_^2^) + (0.0372*P*)^2^ + 1.7501*P*] where *P* = (*F*_o_^2^ + 2*F*_c_^2^)/3
*S* = 1.02	(Δ/σ)_max_ = 0.001
11046 reflections	Δρ_max_ = 0.35 e Å^−^^3^
607 parameters	Δρ_min_ = −0.22 e Å^−^^3^

### Special details

**Table:**

Geometry. All e.s.d.'s (except the e.s.d. in the dihedral angle between two l.s. planes) are estimated using the full covariance matrix. The cell e.s.d.'s are taken into account individually in the estimation of e.s.d.'s in distances, angles and torsion angles; correlations between e.s.d.'s in cell parameters are only used when they are defined by crystal symmetry. An approximate (isotropic) treatment of cell e.s.d.'s is used for estimating e.s.d.'s involving l.s. planes.

### Fractional atomic coordinates and isotropic or equivalent isotropic displacement parameters (Å^2^)

**Table:**

	*x*	*y*	*z*	*U*_iso_*/*U*_eq_	
O1A	−0.26253 (16)	0.40731 (5)	0.53333 (5)	0.0423 (3)	
O2A	−0.02317 (16)	0.36271 (4)	0.51986 (4)	0.0405 (3)	
O3A	0.09946 (11)	0.75766 (4)	0.62417 (3)	0.01910 (18)	
O4A	0.63124 (11)	0.82755 (4)	0.69184 (3)	0.01848 (18)	
O5A	0.58188 (11)	0.87760 (4)	0.52350 (3)	0.02218 (19)	
N1A	−0.10267 (18)	0.40448 (5)	0.53527 (4)	0.0303 (3)	
N2A	0.21581 (12)	0.64878 (4)	0.61833 (4)	0.01462 (19)	
N3A	0.32523 (13)	0.69387 (4)	0.63413 (4)	0.01431 (19)	
H3A	0.437 (2)	0.6869 (7)	0.6411 (6)	0.030 (4)*	
N4A	0.52650 (13)	0.78002 (4)	0.67838 (4)	0.0158 (2)	
C1A	0.18827 (16)	0.55055 (5)	0.59120 (4)	0.0175 (2)	
C2A	0.27370 (18)	0.50151 (6)	0.57471 (5)	0.0233 (3)	
H2AA	0.3977	0.5014	0.5757	0.028*	
C3A	0.17973 (19)	0.45306 (6)	0.55686 (5)	0.0251 (3)	
H3AA	0.2375	0.4198	0.5455	0.030*	
C4A	0.00012 (19)	0.45453 (5)	0.55613 (5)	0.0231 (3)	
C5A	−0.08892 (18)	0.50194 (6)	0.57312 (5)	0.0245 (3)	
H5AA	−0.2127	0.5014	0.5728	0.029*	
C6A	0.00629 (17)	0.55002 (5)	0.59056 (5)	0.0210 (3)	
H6AA	−0.0525	0.5830	0.6022	0.025*	
C7A	0.29202 (15)	0.60136 (5)	0.60856 (4)	0.0167 (2)	
H7AA	0.4161	0.5992	0.6126	0.020*	
C8A	0.25467 (15)	0.74729 (5)	0.63586 (4)	0.0141 (2)	
C9A	0.38222 (15)	0.79508 (5)	0.65171 (4)	0.0141 (2)	
C10A	0.78988 (17)	0.80896 (6)	0.72185 (6)	0.0283 (3)	
H10A	0.8557	0.8426	0.7362	0.042*	
H10B	0.8608	0.7872	0.6986	0.042*	
H10C	0.7615	0.7843	0.7513	0.042*	
C11A	0.32872 (15)	0.85461 (5)	0.63561 (5)	0.0161 (2)	
C12A	0.18674 (16)	0.87956 (6)	0.65749 (5)	0.0227 (3)	
H12A	0.1305	0.8593	0.6837	0.027*	
C13A	0.12772 (17)	0.93369 (6)	0.64103 (6)	0.0287 (3)	
H13A	0.0335	0.9511	0.6568	0.034*	
C14A	0.20638 (18)	0.96246 (6)	0.60155 (6)	0.0285 (3)	
H14A	0.1623	0.9987	0.5890	0.034*	
C15A	0.34927 (18)	0.93837 (5)	0.58034 (5)	0.0234 (3)	
H15A	0.4031	0.9586	0.5536	0.028*	
C16A	0.41512 (16)	0.88485 (5)	0.59776 (5)	0.0172 (2)	
C17A	0.58044 (16)	0.86198 (5)	0.57803 (4)	0.0179 (2)	
H17A	0.6839	0.8785	0.5994	0.022*	
H17B	0.5849	0.8195	0.5818	0.022*	
C18A	0.73512 (16)	0.86995 (5)	0.50045 (5)	0.0180 (2)	
C19A	0.89187 (17)	0.85147 (6)	0.52730 (5)	0.0221 (3)	
H19A	0.8994	0.8430	0.5644	0.027*	
C20A	1.03816 (17)	0.84545 (6)	0.49918 (6)	0.0262 (3)	
H20A	1.1461	0.8330	0.5173	0.031*	
C21A	1.02759 (18)	0.85741 (6)	0.44521 (6)	0.0273 (3)	
H21A	1.1279	0.8536	0.4263	0.033*	
C22A	0.86849 (18)	0.87511 (5)	0.41876 (5)	0.0232 (3)	
H22A	0.8613	0.8827	0.3816	0.028*	
C23A	0.72052 (17)	0.88192 (5)	0.44540 (5)	0.0191 (2)	
C24A	0.54785 (18)	0.89964 (6)	0.41707 (5)	0.0238 (3)	
H24A	0.5586	0.9032	0.3788	0.036*	
H24B	0.5127	0.9368	0.4310	0.036*	
H24C	0.4594	0.8705	0.4229	0.036*	
O1B	0.38132 (18)	0.77311 (5)	0.32876 (4)	0.0468 (3)	
O2B	0.15770 (17)	0.74670 (6)	0.36820 (5)	0.0509 (3)	
O3B	0.62240 (11)	0.63511 (4)	0.68689 (3)	0.01902 (18)	
O4B	1.16718 (11)	0.66162 (4)	0.77511 (3)	0.01737 (17)	
O5B	1.15483 (11)	0.48146 (4)	0.72363 (3)	0.01938 (18)	
N1B	0.31493 (19)	0.75518 (5)	0.36773 (4)	0.0308 (3)	
N2B	0.70567 (12)	0.68705 (4)	0.59787 (4)	0.01368 (19)	
N3B	0.82799 (13)	0.67735 (4)	0.64094 (4)	0.01422 (19)	
H3B	0.939 (2)	0.6897 (6)	0.6404 (6)	0.023 (4)*	
N4B	1.04705 (12)	0.67334 (4)	0.73131 (4)	0.01504 (19)	
C1B	0.64769 (15)	0.71712 (5)	0.50724 (4)	0.0149 (2)	
C2B	0.71807 (17)	0.73457 (5)	0.46063 (5)	0.0211 (3)	
H2BA	0.8413	0.7375	0.4603	0.025*	
C3B	0.60910 (19)	0.74755 (5)	0.41500 (5)	0.0237 (3)	
H3BA	0.6562	0.7596	0.3834	0.028*	
C4B	0.43094 (18)	0.74257 (5)	0.41647 (5)	0.0213 (3)	
C5B	0.35669 (17)	0.72551 (6)	0.46181 (5)	0.0224 (3)	
H5BA	0.2333	0.7226	0.4618	0.027*	
C6B	0.46675 (16)	0.71285 (5)	0.50711 (5)	0.0189 (2)	
H6BA	0.4184	0.7011	0.5386	0.023*	
C7B	0.76688 (15)	0.70484 (5)	0.55520 (4)	0.0151 (2)	
H7BA	0.8894	0.7101	0.5547	0.018*	
C8B	0.77310 (15)	0.65102 (5)	0.68428 (4)	0.0135 (2)	
C9B	0.91295 (14)	0.64002 (5)	0.72976 (4)	0.0135 (2)	
C10B	1.30753 (16)	0.70259 (6)	0.77600 (5)	0.0228 (3)	
H10D	1.3809	0.6998	0.8101	0.034*	
H10E	1.2591	0.7416	0.7719	0.034*	
H10F	1.3784	0.6944	0.7467	0.034*	
C11B	0.87970 (14)	0.59275 (5)	0.76739 (4)	0.0136 (2)	
C12B	0.73387 (15)	0.59676 (5)	0.79640 (5)	0.0168 (2)	
H12B	0.6619	0.6301	0.7932	0.020*	
C13B	0.69440 (16)	0.55214 (6)	0.82974 (5)	0.0193 (2)	
H13B	0.5972	0.5553	0.8501	0.023*	
C14B	0.79674 (16)	0.50289 (5)	0.83340 (5)	0.0197 (2)	
H14B	0.7677	0.4719	0.8556	0.024*	
C15B	0.94123 (16)	0.49860 (5)	0.80488 (5)	0.0174 (2)	
H15B	1.0106	0.4646	0.8076	0.021*	
C16B	0.98577 (15)	0.54373 (5)	0.77224 (4)	0.0141 (2)	
C17B	1.15104 (15)	0.53835 (5)	0.74491 (5)	0.0157 (2)	
H17C	1.1514	0.5671	0.7159	0.019*	
H17D	1.2549	0.5450	0.7708	0.019*	
C18B	1.30751 (16)	0.46297 (5)	0.70551 (4)	0.0173 (2)	
C19B	1.45797 (16)	0.49618 (6)	0.70528 (5)	0.0202 (2)	
H19B	1.4604	0.5345	0.7186	0.024*	
C20B	1.60527 (17)	0.47275 (6)	0.68534 (5)	0.0262 (3)	
H20B	1.7091	0.4950	0.6856	0.031*	
C21B	1.60113 (19)	0.41739 (7)	0.66519 (6)	0.0316 (3)	
H21B	1.7010	0.4017	0.6511	0.038*	
C22B	1.44928 (19)	0.38478 (6)	0.66570 (5)	0.0285 (3)	
H22B	1.4473	0.3467	0.6519	0.034*	
C23B	1.30058 (17)	0.40636 (5)	0.68578 (5)	0.0208 (3)	
C24B	1.13482 (19)	0.37187 (6)	0.68574 (6)	0.0277 (3)	
H24D	1.1497	0.3346	0.6685	0.042*	
H24E	1.0381	0.3929	0.6661	0.042*	
H24F	1.1086	0.3656	0.7226	0.042*	

### Atomic displacement parameters (Å^2^)

**Table:**

	*U*^11^	*U*^22^	*U*^33^	*U*^12^	*U*^13^	*U*^23^
O1A	0.0443 (7)	0.0344 (6)	0.0491 (7)	−0.0186 (5)	0.0097 (5)	−0.0134 (5)
O2A	0.0595 (8)	0.0217 (5)	0.0416 (6)	−0.0068 (5)	0.0120 (5)	−0.0129 (4)
O3A	0.0119 (4)	0.0179 (4)	0.0267 (4)	−0.0008 (3)	−0.0017 (3)	0.0026 (3)
O4A	0.0147 (4)	0.0185 (4)	0.0211 (4)	−0.0050 (3)	−0.0039 (3)	−0.0009 (3)
O5A	0.0194 (4)	0.0303 (5)	0.0167 (4)	0.0004 (4)	0.0012 (3)	0.0059 (3)
N1A	0.0474 (8)	0.0215 (6)	0.0227 (6)	−0.0116 (5)	0.0074 (5)	−0.0043 (4)
N2A	0.0138 (5)	0.0159 (5)	0.0140 (4)	−0.0033 (4)	0.0002 (4)	0.0004 (4)
N3A	0.0093 (5)	0.0158 (5)	0.0175 (5)	−0.0017 (4)	−0.0002 (4)	−0.0010 (4)
N4A	0.0142 (5)	0.0171 (5)	0.0159 (5)	−0.0045 (4)	0.0009 (4)	−0.0011 (4)
C1A	0.0214 (6)	0.0169 (6)	0.0135 (5)	−0.0003 (5)	−0.0017 (4)	−0.0002 (4)
C2A	0.0242 (7)	0.0221 (6)	0.0224 (6)	0.0044 (5)	−0.0038 (5)	−0.0023 (5)
C3A	0.0362 (8)	0.0179 (6)	0.0201 (6)	0.0056 (6)	−0.0029 (5)	−0.0028 (5)
C4A	0.0376 (8)	0.0172 (6)	0.0142 (5)	−0.0070 (5)	0.0016 (5)	−0.0011 (4)
C5A	0.0258 (7)	0.0245 (7)	0.0239 (6)	−0.0065 (5)	0.0052 (5)	−0.0043 (5)
C6A	0.0229 (6)	0.0183 (6)	0.0223 (6)	−0.0023 (5)	0.0044 (5)	−0.0046 (5)
C7A	0.0138 (5)	0.0193 (6)	0.0165 (5)	0.0013 (5)	−0.0006 (4)	−0.0006 (4)
C8A	0.0121 (5)	0.0171 (5)	0.0129 (5)	−0.0018 (4)	0.0012 (4)	0.0019 (4)
C9A	0.0126 (5)	0.0161 (5)	0.0138 (5)	−0.0014 (4)	0.0020 (4)	−0.0002 (4)
C10A	0.0185 (6)	0.0311 (7)	0.0325 (7)	−0.0036 (6)	−0.0113 (5)	0.0013 (6)
C11A	0.0135 (5)	0.0148 (5)	0.0192 (5)	−0.0006 (4)	−0.0032 (4)	−0.0028 (4)
C12A	0.0148 (6)	0.0224 (6)	0.0305 (7)	−0.0012 (5)	0.0016 (5)	−0.0049 (5)
C13A	0.0166 (6)	0.0222 (7)	0.0463 (8)	0.0031 (5)	−0.0014 (6)	−0.0099 (6)
C14A	0.0257 (7)	0.0147 (6)	0.0425 (8)	0.0040 (5)	−0.0097 (6)	−0.0027 (5)
C15A	0.0279 (7)	0.0153 (6)	0.0255 (6)	−0.0016 (5)	−0.0047 (5)	0.0006 (5)
C16A	0.0193 (6)	0.0144 (5)	0.0168 (5)	−0.0024 (5)	−0.0033 (4)	−0.0025 (4)
C17A	0.0225 (6)	0.0152 (5)	0.0159 (5)	−0.0013 (5)	0.0015 (4)	0.0016 (4)
C18A	0.0186 (6)	0.0159 (6)	0.0192 (6)	−0.0037 (5)	0.0012 (4)	0.0006 (4)
C19A	0.0227 (6)	0.0229 (6)	0.0199 (6)	−0.0030 (5)	−0.0024 (5)	0.0005 (5)
C20A	0.0181 (6)	0.0276 (7)	0.0319 (7)	−0.0028 (5)	−0.0028 (5)	−0.0036 (5)
C21A	0.0228 (7)	0.0281 (7)	0.0323 (7)	−0.0045 (6)	0.0087 (5)	−0.0040 (6)
C22A	0.0299 (7)	0.0203 (6)	0.0202 (6)	−0.0053 (5)	0.0058 (5)	0.0008 (5)
C23A	0.0235 (6)	0.0138 (5)	0.0197 (6)	−0.0043 (5)	0.0009 (5)	0.0019 (4)
C24A	0.0285 (7)	0.0242 (6)	0.0178 (6)	−0.0007 (5)	−0.0018 (5)	0.0050 (5)
O1B	0.0826 (9)	0.0361 (6)	0.0180 (5)	−0.0131 (6)	−0.0128 (5)	0.0090 (4)
O2B	0.0429 (7)	0.0751 (9)	0.0307 (6)	0.0191 (7)	−0.0162 (5)	−0.0011 (6)
O3B	0.0124 (4)	0.0228 (4)	0.0212 (4)	−0.0031 (3)	−0.0016 (3)	0.0059 (3)
O4B	0.0134 (4)	0.0196 (4)	0.0177 (4)	−0.0032 (3)	−0.0056 (3)	0.0028 (3)
O5B	0.0167 (4)	0.0159 (4)	0.0262 (4)	0.0012 (3)	0.0048 (3)	−0.0049 (3)
N1B	0.0534 (8)	0.0185 (5)	0.0175 (5)	0.0063 (5)	−0.0112 (5)	−0.0022 (4)
N2B	0.0113 (4)	0.0140 (5)	0.0149 (4)	0.0010 (4)	−0.0026 (3)	−0.0001 (3)
N3B	0.0088 (4)	0.0176 (5)	0.0157 (5)	−0.0002 (4)	−0.0018 (3)	0.0005 (4)
N4B	0.0127 (5)	0.0172 (5)	0.0144 (4)	0.0015 (4)	−0.0029 (4)	−0.0001 (4)
C1B	0.0189 (6)	0.0112 (5)	0.0145 (5)	−0.0018 (4)	0.0009 (4)	−0.0015 (4)
C2B	0.0243 (6)	0.0217 (6)	0.0178 (6)	−0.0082 (5)	0.0044 (5)	−0.0026 (5)
C3B	0.0396 (8)	0.0178 (6)	0.0139 (5)	−0.0088 (6)	0.0035 (5)	−0.0009 (4)
C4B	0.0362 (7)	0.0121 (5)	0.0137 (5)	0.0020 (5)	−0.0064 (5)	−0.0013 (4)
C5B	0.0201 (6)	0.0247 (6)	0.0214 (6)	0.0033 (5)	−0.0029 (5)	−0.0007 (5)
C6B	0.0184 (6)	0.0223 (6)	0.0159 (5)	0.0005 (5)	0.0013 (4)	0.0030 (4)
C7B	0.0119 (5)	0.0162 (5)	0.0172 (5)	−0.0018 (4)	0.0017 (4)	−0.0022 (4)
C8B	0.0122 (5)	0.0112 (5)	0.0167 (5)	0.0020 (4)	−0.0009 (4)	−0.0010 (4)
C9B	0.0116 (5)	0.0131 (5)	0.0155 (5)	0.0022 (4)	0.0001 (4)	−0.0012 (4)
C10B	0.0177 (6)	0.0265 (7)	0.0229 (6)	−0.0095 (5)	−0.0049 (5)	0.0022 (5)
C11B	0.0126 (5)	0.0150 (5)	0.0124 (5)	−0.0012 (4)	−0.0024 (4)	−0.0009 (4)
C12B	0.0134 (5)	0.0178 (6)	0.0187 (5)	0.0016 (5)	−0.0009 (4)	−0.0021 (4)
C13B	0.0139 (6)	0.0258 (6)	0.0186 (6)	−0.0029 (5)	0.0027 (4)	−0.0006 (5)
C14B	0.0186 (6)	0.0212 (6)	0.0191 (6)	−0.0049 (5)	0.0002 (5)	0.0045 (5)
C15B	0.0167 (6)	0.0154 (5)	0.0193 (6)	0.0011 (5)	−0.0014 (4)	0.0012 (4)
C16B	0.0132 (5)	0.0156 (5)	0.0127 (5)	−0.0006 (4)	−0.0018 (4)	−0.0016 (4)
C17B	0.0157 (5)	0.0136 (5)	0.0177 (5)	0.0014 (4)	0.0012 (4)	−0.0012 (4)
C18B	0.0180 (6)	0.0190 (6)	0.0145 (5)	0.0053 (5)	−0.0002 (4)	0.0004 (4)
C19B	0.0188 (6)	0.0234 (6)	0.0179 (6)	0.0019 (5)	0.0001 (5)	−0.0018 (5)
C20B	0.0164 (6)	0.0392 (8)	0.0226 (6)	0.0023 (6)	0.0002 (5)	−0.0034 (5)
C21B	0.0223 (7)	0.0431 (9)	0.0296 (7)	0.0138 (6)	0.0040 (5)	−0.0054 (6)
C22B	0.0320 (7)	0.0250 (7)	0.0279 (7)	0.0123 (6)	−0.0003 (6)	−0.0060 (5)
C23B	0.0245 (6)	0.0189 (6)	0.0183 (6)	0.0051 (5)	−0.0014 (5)	0.0000 (4)
C24B	0.0335 (8)	0.0172 (6)	0.0325 (7)	−0.0005 (6)	0.0029 (6)	−0.0032 (5)

### Geometric parameters (Å, º)

**Table:**

O1A—N1A	1.2258 (17)	O1B—N1B	1.2242 (17)
O2A—N1A	1.2296 (16)	O2B—N1B	1.2250 (18)
O3A—C8A	1.2228 (14)	O3B—C8B	1.2233 (14)
O4A—N4A	1.3873 (13)	O4B—N4B	1.3918 (12)
O4A—C10A	1.4341 (15)	O4B—C10B	1.4361 (14)
O5A—C18A	1.3758 (15)	O5B—C18B	1.3700 (14)
O5A—C17A	1.4222 (14)	O5B—C17B	1.4266 (14)
N1A—C4A	1.4716 (17)	N1B—C4B	1.4719 (16)
N2A—C7A	1.2823 (15)	N2B—C7B	1.2844 (15)
N2A—N3A	1.3759 (14)	N2B—N3B	1.3803 (13)
N3A—C8A	1.3560 (15)	N3B—C8B	1.3552 (15)
N3A—H3A	0.871 (17)	N3B—H3B	0.901 (16)
N4A—C9A	1.2853 (15)	N4B—C9B	1.2856 (15)
C1A—C6A	1.3965 (17)	C1B—C6B	1.3932 (17)
C1A—C2A	1.3982 (17)	C1B—C2B	1.4013 (16)
C1A—C7A	1.4654 (17)	C1B—C7B	1.4683 (16)
C2A—C3A	1.3865 (19)	C2B—C3B	1.3852 (18)
C2A—H2AA	0.9500	C2B—H2BA	0.9500
C3A—C4A	1.378 (2)	C3B—C4B	1.378 (2)
C3A—H3AA	0.9500	C3B—H3BA	0.9500
C4A—C5A	1.3864 (19)	C4B—C5B	1.3852 (18)
C5A—C6A	1.3815 (18)	C5B—C6B	1.3816 (17)
C5A—H5AA	0.9500	C5B—H5BA	0.9500
C6A—H6AA	0.9500	C6B—H6BA	0.9500
C7A—H7AA	0.9500	C7B—H7BA	0.9500
C8A—C9A	1.5072 (16)	C8B—C9B	1.5110 (15)
C9A—C11A	1.4864 (16)	C9B—C11B	1.4892 (16)
C10A—H10A	0.9800	C10B—H10D	0.9800
C10A—H10B	0.9800	C10B—H10E	0.9800
C10A—H10C	0.9800	C10B—H10F	0.9800
C11A—C12A	1.3965 (17)	C11B—C16B	1.3979 (16)
C11A—C16A	1.4039 (17)	C11B—C12B	1.4012 (16)
C12A—C13A	1.3851 (19)	C12B—C13B	1.3865 (17)
C12A—H12A	0.9500	C12B—H12B	0.9500
C13A—C14A	1.386 (2)	C13B—C14B	1.3854 (18)
C13A—H13A	0.9500	C13B—H13B	0.9500
C14A—C15A	1.386 (2)	C14B—C15B	1.3855 (17)
C14A—H14A	0.9500	C14B—H14B	0.9500
C15A—C16A	1.3954 (17)	C15B—C16B	1.3956 (16)
C15A—H15A	0.9500	C15B—H15B	0.9500
C16A—C17A	1.5065 (17)	C16B—C17B	1.5094 (16)
C17A—H17A	0.9900	C17B—H17C	0.9900
C17A—H17B	0.9900	C17B—H17D	0.9900
C18A—C19A	1.3881 (18)	C18B—C19B	1.3899 (18)
C18A—C23A	1.4076 (16)	C18B—C23B	1.4040 (17)
C19A—C20A	1.3947 (19)	C19B—C20B	1.3941 (18)
C19A—H19A	0.9500	C19B—H19B	0.9500
C20A—C21A	1.382 (2)	C20B—C21B	1.381 (2)
C20A—H20A	0.9500	C20B—H20B	0.9500
C21A—C22A	1.394 (2)	C21B—C22B	1.392 (2)
C21A—H21A	0.9500	C21B—H21B	0.9500
C22A—C23A	1.3863 (18)	C22B—C23B	1.3888 (18)
C22A—H22A	0.9500	C22B—H22B	0.9500
C23A—C24A	1.4989 (18)	C23B—C24B	1.5040 (19)
C24A—H24A	0.9800	C24B—H24D	0.9800
C24A—H24B	0.9800	C24B—H24E	0.9800
C24A—H24C	0.9800	C24B—H24F	0.9800
			
N4A—O4A—C10A	109.30 (9)	N4B—O4B—C10B	108.64 (8)
C18A—O5A—C17A	117.74 (9)	C18B—O5B—C17B	117.78 (9)
O1A—N1A—O2A	123.86 (12)	O1B—N1B—O2B	123.61 (12)
O1A—N1A—C4A	118.11 (12)	O1B—N1B—C4B	118.07 (13)
O2A—N1A—C4A	118.01 (13)	O2B—N1B—C4B	118.32 (12)
C7A—N2A—N3A	115.55 (10)	C7B—N2B—N3B	115.65 (10)
C8A—N3A—N2A	118.07 (10)	C8B—N3B—N2B	117.68 (10)
C8A—N3A—H3A	123.4 (11)	C8B—N3B—H3B	121.9 (9)
N2A—N3A—H3A	118.4 (11)	N2B—N3B—H3B	120.4 (9)
C9A—N4A—O4A	111.07 (9)	C9B—N4B—O4B	111.82 (9)
C6A—C1A—C2A	119.26 (11)	C6B—C1B—C2B	119.21 (11)
C6A—C1A—C7A	121.62 (11)	C6B—C1B—C7B	121.81 (10)
C2A—C1A—C7A	119.12 (11)	C2B—C1B—C7B	118.97 (11)
C3A—C2A—C1A	120.81 (12)	C3B—C2B—C1B	120.43 (12)
C3A—C2A—H2AA	119.6	C3B—C2B—H2BA	119.8
C1A—C2A—H2AA	119.6	C1B—C2B—H2BA	119.8
C4A—C3A—C2A	118.16 (12)	C4B—C3B—C2B	118.56 (11)
C4A—C3A—H3AA	120.9	C4B—C3B—H3BA	120.7
C2A—C3A—H3AA	120.9	C2B—C3B—H3BA	120.7
C3A—C4A—C5A	122.68 (12)	C3B—C4B—C5B	122.63 (11)
C3A—C4A—N1A	119.05 (12)	C3B—C4B—N1B	118.70 (12)
C5A—C4A—N1A	118.26 (13)	C5B—C4B—N1B	118.67 (12)
C6A—C5A—C4A	118.54 (13)	C6B—C5B—C4B	118.26 (12)
C6A—C5A—H5AA	120.7	C6B—C5B—H5BA	120.9
C4A—C5A—H5AA	120.7	C4B—C5B—H5BA	120.9
C5A—C6A—C1A	120.53 (12)	C5B—C6B—C1B	120.92 (11)
C5A—C6A—H6AA	119.7	C5B—C6B—H6BA	119.5
C1A—C6A—H6AA	119.7	C1B—C6B—H6BA	119.5
N2A—C7A—C1A	120.21 (11)	N2B—C7B—C1B	120.04 (10)
N2A—C7A—H7AA	119.9	N2B—C7B—H7BA	120.0
C1A—C7A—H7AA	119.9	C1B—C7B—H7BA	120.0
O3A—C8A—N3A	123.75 (11)	O3B—C8B—N3B	123.56 (10)
O3A—C8A—C9A	120.72 (10)	O3B—C8B—C9B	120.83 (10)
N3A—C8A—C9A	115.50 (10)	N3B—C8B—C9B	115.58 (10)
N4A—C9A—C11A	126.56 (10)	N4B—C9B—C11B	127.77 (10)
N4A—C9A—C8A	116.19 (10)	N4B—C9B—C8B	115.32 (10)
C11A—C9A—C8A	117.24 (10)	C11B—C9B—C8B	116.91 (10)
O4A—C10A—H10A	109.5	O4B—C10B—H10D	109.5
O4A—C10A—H10B	109.5	O4B—C10B—H10E	109.5
H10A—C10A—H10B	109.5	H10D—C10B—H10E	109.5
O4A—C10A—H10C	109.5	O4B—C10B—H10F	109.5
H10A—C10A—H10C	109.5	H10D—C10B—H10F	109.5
H10B—C10A—H10C	109.5	H10E—C10B—H10F	109.5
C12A—C11A—C16A	120.16 (11)	C16B—C11B—C12B	119.89 (10)
C12A—C11A—C9A	118.91 (11)	C16B—C11B—C9B	121.31 (10)
C16A—C11A—C9A	120.90 (10)	C12B—C11B—C9B	118.74 (10)
C13A—C12A—C11A	120.22 (12)	C13B—C12B—C11B	120.11 (11)
C13A—C12A—H12A	119.9	C13B—C12B—H12B	119.9
C11A—C12A—H12A	119.9	C11B—C12B—H12B	119.9
C12A—C13A—C14A	119.90 (13)	C14B—C13B—C12B	119.98 (11)
C12A—C13A—H13A	120.0	C14B—C13B—H13B	120.0
C14A—C13A—H13A	120.0	C12B—C13B—H13B	120.0
C13A—C14A—C15A	120.12 (12)	C15B—C14B—C13B	120.25 (11)
C13A—C14A—H14A	119.9	C15B—C14B—H14B	119.9
C15A—C14A—H14A	119.9	C13B—C14B—H14B	119.9
C14A—C15A—C16A	120.92 (12)	C14B—C15B—C16B	120.58 (11)
C14A—C15A—H15A	119.5	C14B—C15B—H15B	119.7
C16A—C15A—H15A	119.5	C16B—C15B—H15B	119.7
C15A—C16A—C11A	118.54 (11)	C15B—C16B—C11B	119.15 (11)
C15A—C16A—C17A	120.06 (11)	C15B—C16B—C17B	118.32 (10)
C11A—C16A—C17A	121.34 (10)	C11B—C16B—C17B	122.50 (10)
O5A—C17A—C16A	108.55 (10)	O5B—C17B—C16B	107.34 (9)
O5A—C17A—H17A	110.0	O5B—C17B—H17C	110.2
C16A—C17A—H17A	110.0	C16B—C17B—H17C	110.2
O5A—C17A—H17B	110.0	O5B—C17B—H17D	110.2
C16A—C17A—H17B	110.0	C16B—C17B—H17D	110.2
H17A—C17A—H17B	108.4	H17C—C17B—H17D	108.5
O5A—C18A—C19A	124.84 (11)	O5B—C18B—C19B	124.60 (11)
O5A—C18A—C23A	113.83 (11)	O5B—C18B—C23B	114.10 (11)
C19A—C18A—C23A	121.32 (11)	C19B—C18B—C23B	121.30 (11)
C18A—C19A—C20A	119.16 (12)	C18B—C19B—C20B	119.39 (12)
C18A—C19A—H19A	120.4	C18B—C19B—H19B	120.3
C20A—C19A—H19A	120.4	C20B—C19B—H19B	120.3
C21A—C20A—C19A	120.62 (13)	C21B—C20B—C19B	120.41 (13)
C21A—C20A—H20A	119.7	C21B—C20B—H20B	119.8
C19A—C20A—H20A	119.7	C19B—C20B—H20B	119.8
C20A—C21A—C22A	119.41 (12)	C20B—C21B—C22B	119.40 (12)
C20A—C21A—H21A	120.3	C20B—C21B—H21B	120.3
C22A—C21A—H21A	120.3	C22B—C21B—H21B	120.3
C23A—C22A—C21A	121.64 (12)	C23B—C22B—C21B	121.88 (13)
C23A—C22A—H22A	119.2	C23B—C22B—H22B	119.1
C21A—C22A—H22A	119.2	C21B—C22B—H22B	119.1
C22A—C23A—C18A	117.83 (12)	C22B—C23B—C18B	117.61 (12)
C22A—C23A—C24A	122.02 (11)	C22B—C23B—C24B	122.36 (12)
C18A—C23A—C24A	120.12 (11)	C18B—C23B—C24B	120.02 (11)
C23A—C24A—H24A	109.5	C23B—C24B—H24D	109.5
C23A—C24A—H24B	109.5	C23B—C24B—H24E	109.5
H24A—C24A—H24B	109.5	H24D—C24B—H24E	109.5
C23A—C24A—H24C	109.5	C23B—C24B—H24F	109.5
H24A—C24A—H24C	109.5	H24D—C24B—H24F	109.5
H24B—C24A—H24C	109.5	H24E—C24B—H24F	109.5
			
C7A—N2A—N3A—C8A	168.52 (10)	C7B—N2B—N3B—C8B	−170.95 (10)
C10A—O4A—N4A—C9A	179.55 (10)	C10B—O4B—N4B—C9B	−176.53 (10)
C6A—C1A—C2A—C3A	1.25 (18)	C6B—C1B—C2B—C3B	0.15 (18)
C7A—C1A—C2A—C3A	−178.53 (11)	C7B—C1B—C2B—C3B	−178.76 (11)
C1A—C2A—C3A—C4A	−0.35 (19)	C1B—C2B—C3B—C4B	−0.39 (18)
C2A—C3A—C4A—C5A	−0.91 (19)	C2B—C3B—C4B—C5B	0.46 (19)
C2A—C3A—C4A—N1A	177.51 (11)	C2B—C3B—C4B—N1B	−178.76 (11)
O1A—N1A—C4A—C3A	−177.67 (12)	O1B—N1B—C4B—C3B	−4.50 (17)
O2A—N1A—C4A—C3A	0.95 (18)	O2B—N1B—C4B—C3B	174.85 (13)
O1A—N1A—C4A—C5A	0.82 (18)	O1B—N1B—C4B—C5B	176.25 (12)
O2A—N1A—C4A—C5A	179.44 (12)	O2B—N1B—C4B—C5B	−4.41 (18)
C3A—C4A—C5A—C6A	1.21 (19)	C3B—C4B—C5B—C6B	−0.27 (19)
N1A—C4A—C5A—C6A	−177.22 (11)	N1B—C4B—C5B—C6B	178.95 (11)
C4A—C5A—C6A—C1A	−0.26 (19)	C4B—C5B—C6B—C1B	0.01 (19)
C2A—C1A—C6A—C5A	−0.93 (18)	C2B—C1B—C6B—C5B	0.05 (18)
C7A—C1A—C6A—C5A	178.84 (11)	C7B—C1B—C6B—C5B	178.92 (11)
N3A—N2A—C7A—C1A	−179.72 (9)	N3B—N2B—C7B—C1B	−179.29 (10)
C6A—C1A—C7A—N2A	−7.33 (17)	C6B—C1B—C7B—N2B	3.31 (17)
C2A—C1A—C7A—N2A	172.45 (11)	C2B—C1B—C7B—N2B	−177.82 (11)
N2A—N3A—C8A—O3A	0.00 (16)	N2B—N3B—C8B—O3B	0.70 (17)
N2A—N3A—C8A—C9A	−178.06 (9)	N2B—N3B—C8B—C9B	178.48 (9)
O4A—N4A—C9A—C11A	0.81 (16)	O4B—N4B—C9B—C11B	−1.30 (16)
O4A—N4A—C9A—C8A	−178.51 (9)	O4B—N4B—C9B—C8B	178.23 (9)
O3A—C8A—C9A—N4A	159.45 (11)	O3B—C8B—C9B—N4B	−158.18 (11)
N3A—C8A—C9A—N4A	−22.43 (14)	N3B—C8B—C9B—N4B	23.97 (14)
O3A—C8A—C9A—C11A	−19.94 (15)	O3B—C8B—C9B—C11B	21.41 (15)
N3A—C8A—C9A—C11A	158.18 (10)	N3B—C8B—C9B—C11B	−156.45 (10)
N4A—C9A—C11A—C12A	−113.93 (14)	N4B—C9B—C11B—C16B	−64.63 (16)
C8A—C9A—C11A—C12A	65.38 (14)	C8B—C9B—C11B—C16B	115.84 (12)
N4A—C9A—C11A—C16A	68.15 (16)	N4B—C9B—C11B—C12B	118.44 (13)
C8A—C9A—C11A—C16A	−112.54 (12)	C8B—C9B—C11B—C12B	−61.09 (14)
C16A—C11A—C12A—C13A	1.55 (18)	C16B—C11B—C12B—C13B	−0.20 (17)
C9A—C11A—C12A—C13A	−176.39 (11)	C9B—C11B—C12B—C13B	176.77 (10)
C11A—C12A—C13A—C14A	2.0 (2)	C11B—C12B—C13B—C14B	−1.59 (18)
C12A—C13A—C14A—C15A	−3.1 (2)	C12B—C13B—C14B—C15B	1.63 (18)
C13A—C14A—C15A—C16A	0.6 (2)	C13B—C14B—C15B—C16B	0.12 (18)
C14A—C15A—C16A—C11A	2.83 (18)	C14B—C15B—C16B—C11B	−1.89 (17)
C14A—C15A—C16A—C17A	−174.24 (12)	C14B—C15B—C16B—C17B	176.07 (11)
C12A—C11A—C16A—C15A	−3.91 (17)	C12B—C11B—C16B—C15B	1.92 (16)
C9A—C11A—C16A—C15A	173.99 (11)	C9B—C11B—C16B—C15B	−174.98 (10)
C12A—C11A—C16A—C17A	173.11 (11)	C12B—C11B—C16B—C17B	−175.95 (10)
C9A—C11A—C16A—C17A	−8.98 (17)	C9B—C11B—C16B—C17B	7.15 (16)
C18A—O5A—C17A—C16A	168.60 (10)	C18B—O5B—C17B—C16B	−168.30 (9)
C15A—C16A—C17A—O5A	−37.34 (15)	C15B—C16B—C17B—O5B	44.51 (13)
C11A—C16A—C17A—O5A	145.68 (11)	C11B—C16B—C17B—O5B	−137.60 (10)
C17A—O5A—C18A—C19A	−4.23 (17)	C17B—O5B—C18B—C19B	−1.51 (16)
C17A—O5A—C18A—C23A	174.92 (10)	C17B—O5B—C18B—C23B	179.35 (10)
O5A—C18A—C19A—C20A	179.98 (12)	O5B—C18B—C19B—C20B	−179.43 (11)
C23A—C18A—C19A—C20A	0.89 (19)	C23B—C18B—C19B—C20B	−0.35 (18)
C18A—C19A—C20A—C21A	−0.3 (2)	C18B—C19B—C20B—C21B	1.04 (19)
C19A—C20A—C21A—C22A	−0.6 (2)	C19B—C20B—C21B—C22B	−1.0 (2)
C20A—C21A—C22A—C23A	1.0 (2)	C20B—C21B—C22B—C23B	0.2 (2)
C21A—C22A—C23A—C18A	−0.43 (19)	C21B—C22B—C23B—C18B	0.44 (19)
C21A—C22A—C23A—C24A	−178.58 (12)	C21B—C22B—C23B—C24B	179.00 (13)
O5A—C18A—C23A—C22A	−179.71 (11)	O5B—C18B—C23B—C22B	178.79 (11)
C19A—C18A—C23A—C22A	−0.52 (18)	C19B—C18B—C23B—C22B	−0.38 (18)
O5A—C18A—C23A—C24A	−1.52 (16)	O5B—C18B—C23B—C24B	0.20 (16)
C19A—C18A—C23A—C24A	177.66 (12)	C19B—C18B—C23B—C24B	−178.97 (11)

### Hydrogen-bond geometry (Å, º)

**Table:**

*D*—H···*A*	*D*—H	H···*A*	*D*···*A*	*D*—H···*A*
N3*A*—H3*A*···O3*B*	0.871 (17)	2.117 (17)	2.8679 (13)	144.0 (14)
N3*A*—H3*A*···N2*B*	0.871 (17)	2.432 (16)	3.1530 (14)	140.5 (13)
C7*A*—H7*AA*···O3*B*	0.95	2.47	3.1523 (14)	129
C10*A*—H10*C*···O1*B*^i^	0.98	2.46	3.3159 (18)	145
C17*A*—H17*B*···N4*A*	0.99	2.68	3.2243 (15)	115
N3*B*—H3*B*···O3*A*^ii^	0.901 (16)	2.068 (16)	2.8605 (13)	146.1 (13)
N3*B*—H3*B*···N2*A*^ii^	0.901 (16)	2.444 (15)	3.1598 (14)	136.6 (12)
C5*B*—H5*BA*···O2*A*^iii^	0.95	2.63	3.3484 (18)	133
C7*B*—H7*BA*···O3*A*^ii^	0.95	2.51	3.1864 (14)	128
C17*B*—H17*C*···N4*B*	0.99	2.63	3.2436 (15)	120

Symmetry codes: (i) *x*+1/2, −*y*+3/2, *z*+1/2; (ii) *x*+1, *y*, *z*; (iii) −*x*, −*y*+1, −*z*+1.
